# Canine atopic dermatitis: validation of recorded diagnosis against practice records in 335 insured Swedish dogs

**DOI:** 10.1186/1751-0147-48-8

**Published:** 2006-06-15

**Authors:** Ane Nødtvedt, Kerstin Bergvall, Ulf Emanuelson, Agneta Egenvall

**Affiliations:** 1 Department of Small Animal Clinical Sciences, Faculty of Veterinary Medicine and Animal Science, Swedish University of Agricultural Sciences, Uppsala, Sweden; 2 Department of Clinical Sciences, Faculty of Veterinary Medicine and Animal Science, Swedish University of Agricultural Sciences, Uppsala, Sweden

## Abstract

A cross-sectional study of insured Swedish dogs with a recorded diagnosis of canine atopic dermatitis (CAD) was performed. In order to validate the correctness of this specific diagnosis in the insurance database, medical records were requested by mail from the attending veterinarians. All dogs with a reimbursed claim for the disease during 2002 were included in the original study sample (n = 373). Medical records were available for 335 individuals (response rate: 89.8%). By scrutinizing the submitted records it was determined that all dogs had been treated for dermatologic disease, and that 327 (97.6%) could be considered to have some allergic skin disease. However, as information regarding dietary trial testing was missing in many dogs the number that were truly atopic could not be determined. The clinical presentation and nature of test diet for dogs with or without response to dietary trial testing was compared for a subset of 109 individuals that had undergone such testing. The only significant difference between these two groups was that the proportion of dogs with reported gastrointestinal signs was higher in the group that subsequently responded to a diet trial. In conclusion, the agreement between the recorded diagnosis in the insurance database and the clinical manifestations recorded in the submitted medical records was considered acceptable. The concern was raised that many attending veterinarians did not exclude cutaneous adverse food reactions before making the diagnosis of CAD.

## Introduction

Canine atopic dermatitis (CAD) is a genetically-predisposed inflammatory and pruritic allergic skin disease, most commonly associated with IgE antibodies to environmental allergens [1]. Among humans, the prevalence of allergic diseases has been increasing in industrialized countries over the past decades, and this has been related to factors regarding lifestyle and environment [2]. Many similarities exist between human and canine atopy, and it is of interest to investigate whether this increase is seen among dogs as well. The available knowledge on the epidemiology of CAD is limited, and more studies within this area are in demand according to the International Task Force on Canine Atopic Dermatitis [3].

At present, no definite diagnostic test exists for CAD and the diagnosis is based upon a typical clinical presentation after the exclusion of plausible differential diagnoses [4]. The main clinical sign of CAD is pruritus, particularly of the face, ears, paws, extremities and/or ventrum [5]. Hence, before a diagnosis is made it is important to rule out other pruritic diseases like flea allergy dermatitis, sarcoptic mange and other ectoparasites, bacterial pyoderma, *Malassezia *dermatitis and cutaneous adverse food reactions (AFR). A set of major and minor criteria for CAD has been proposed, out of which a patient needs to fulfil at least three in each group to be considered having CAD [6]. However, it must be kept in mind that the manifestations of CAD are highly variable between individuals and a patient that fails to fulfil these criteria may still actually be atopic. Also, the criteria proposed by Willemse (1986) [6] have not been properly evaluated regarding sensitivity and specificity and other authors have proposed different criteria [4]. Due to these uncertainties regarding the diagnosing of CAD, it is paramount that individuals included in studies regarding epidemiology, aetiology, therapy or diagnosis of the disease are properly characterized in order to ensure that the diagnosis is correct and to enable comparisons between studies.

Secondary databases, ie that include information not originally collected for research purposes, are commonly used within the field of human epidemiology. Insurance claims records are examples of secondary databases and the limitations of the information included in such registries need to be kept in mind when research is performed [7]. Specifically, the quality of the information coded in a secondary database needs to be addressed, focusing on correctness as well as completeness of information. Correctness can be defined as the proportion of recorded cases that actually have the disease in question, while completeness can be viewed as the proportion of subjects with the disease of interest that are registered in the database [8]. Furthermore, Jordan and others (2004) [8] lets external validity refer to whether the clinician makes the correct diagnosis or not, and internal validity to whether the patient is coded in the database with the diagnosis believed to be correct by the clinician. Various techniques have been applied in studies aiming at validating information in databases of health records. Some recent examples include retrospective collection of relevant clinical reports to be scrutinized by external experts [9], use of external laboratory data for case identification and confirmation [10], and distribution of questionnaires to practitioners to validate a diagnosis in a large human health database [11].

In Sweden, the largest insurance company to offer insurance for dogs has been estimated to cover approximately 30% of the entire canine population [12]. Several studies have been published in which the claims database from this insurance company has been used to study both general and specific morbidity and mortality among Swedish dogs [13,14]. A study into the epidemiology of CAD among insured Swedish dogs revealed that age, breed, habitat, region and season of birth were factors which influenced the incidence rate of the disease [15]. The study also showed an apparent increase in the incidence of CAD through time from 1995 to 2002 and a trend towards affected individuals from high-risk breeds dying at a higher rate than non-affected dogs from the same breeds. The accuracy of diagnostic information in the insurance database was considered reasonable when validated against randomly selected medical records [16]. However, the correctness of the specific diagnosis of CAD has not previously been evaluated in this material and is unknown. A validation of the diagnosis in the insurance claims database is needed as part of a larger project focusing on the epidemiology of CAD among Swedish dogs.

The main objective of this study was to validate the diagnosis of CAD in a large animal insurance database against practice records. The reported clinical presentations were compared between individuals who were atopic only and dogs with a dual diagnosis of atopy and adverse food reactions.

## Materials and methods

### Study population

The Agria insurance company offers two main kinds of insurance for pets. If an animal is covered by the insurance plan for veterinary care, the cost of veterinary treatment is reimbursed to the owner in cases of disease (minus a set deductible). Pets can also be covered by a life-insurance plan, in which case the owner will be reimbursed the monetary value of the pet (as defined through the plan) if the animal dies or is euthanized because of disease or accidents before the age of ten years. The insurance process has been described in detail by Bonnett and others (1997) [17] as well as Egenvall and others (2000) [18].

All reimbursed claims for canine atopic dermatitis during 2002 were extracted from the insurance database, including both life-insurance and veterinary care claims. The resulting file included the following information for each CAD claim: date of claim, claim number, and gender, breed and date of birth of the animal. Information about the attending practitioner and medical record number, as well as the name of the owner and the dog was obtained from the insurance company's claims archive. Letters were sent to attending veterinarians in which they were asked to submit all medical records for the selected cases as identified by pet name, breed, date of birth, record number, date of visit, diagnosis and owner name. The letter did not inform clinicians about the specific aim of the study, only that dogs with reimbursed claims for skin disease were being evaluated. Large practices that contributed more than 10 cases in this material were contacted by telephone prior to the posting. Reminders were sent to veterinarians that had not provided medical records within six weeks of the first posting.

### Descriptive statistics

The principal investigator scrutinized all medical records and recorded the reported clinical signs and any diagnostic tests performed. Each patient was re-classified as atopic or not based upon these clinical findings utilizing a revised version of Willemse's diagnostic criteria. Three or more of the following criteria had to be fulfilled after the exclusion of other pruritic skin disorders in order for an animal to be re-classified as atopic: facial pruritus, paw pruritus, pruritic otitis, pruritic ventral dermatitis, pruritic generalized dermatitis, bilateral interdigital erythema, debut before three years of age, peri -oral, -otic or -ophthalmic erythema, and/or recurring dermatitis for more than two years. Sarcoptic mange was ruled out either by serology or diagnostic treatment. Bacterial pyoderma and *Malassezia *dermatitis was ruled out by cytology and/or treatment of affected individuals. The recorded diagnosis was graded on an eight point scale ranging from (1) "Atopic grade 1, without AFR" to (8) "Other skin diseases" by the investigator. For the definition of each level on the graded scale, see table 1.

**Table 1:** Criteria for re-classification of dogs with a reimbursed claim for CAD based on information from medical records.

**Group**	**Classification**	**Diagnostic criteria**
1	Atopic, grade 1	-Clinical criteria of canine atopic dermatitis fulfilled* -Positive reaction to aero-allergens by use of intradermal skin test or serology testing for specific IgE **-No **response to dietary trial with novel protein source (or hydrolyzed protein)
2	Atopic, grade 2	-Clinical criteria of canine atopic dermatitis fulfilled* -Positive reaction to aero-allergens by use of intradermal skin test or serology testing for specific IgE **-Partial **response to dietary trial with novel protein source (or hydrolyzed protein)
3	Questionable atopic	-Clinical criteria of canine atopic dermatitis fulfilled* -Positive reaction to aero-allergens by use of intradermal skin test or serology testing for specific IgE -Dietary trial **not performed**
4	Plausible atopic	-Successful allergen-specific immunotherapy -Incomplete information about symptoms and/or diagnostic testing
5	Possible atopic	-Clinical criteria of canine atopic dermatitis fulfilled* -No reaction to aero-allergens by use of intradermal skin test, serology testing for specific IgE not performed **-No **response to dietary trial with novel protein source (or hydrolyzed protein)
6	Allergic dermatitis	-Clinical criteria of canine atopic dermatitis fulfilled* -No intradermal skin test, IgE test or elimination diet performed or negative skin test, no elimination diet
7	Cutaneous adverse food reaction (AFR)	-Clinical criteria of canine atopic dermatitis fulfilled* -Complete response to dietary trial with novel protein source (or hydrolyzed protein)
8	Other skin	-Pruritus due to parasites or other skin diseases

* Pruritic patient fulfilling three or more of the following clinical criteria: facial pruritus, paw pruritus, pruritic otitis, pruritic ventral dermatitis, pruritic generalized dermatitis, bilateral interdigital erythema, debut before three years of age, peri- oral, otic or ophthalmic erythema, and/or recurring dermatitis for more than two years; signs persistent after exclusion of ectoparasites, pyodermia and Malazessia dermatitis.

Initially, individuals classified as (1) "Atopic grade 1 – without AFR", (2) "Atopic grade 2 – with AFR", (3) "Questionable atopic", (4) "Plausible atopic" and (5) "Possible atopic" were all re-classified as being CAD positive. A more conservative estimate of the proportion CAD positive dogs was obtained by assuming that all individuals in group 3 would respond completely to a diet trial, and hence were CAD negative. Information about the subgroup of dogs classified as "Atopic, grade 1 – without AFR" and "Possible atopic" was compared to dogs in the groups "Atopic grade 2 – with AFR" and "Adverse food reaction" with respect to; gender, breed, age at debut, type of test diet and recorded clinical signs. The association between belonging to the group "Atopic grade 1" or "Possible atopic" and each categorical variable were tested using a chi-squared test. The difference between debut age in month between the two groups was tested using a students t-test. The purpose of this exercise was to determine whether any of the recorded clinical signs were associated with the response to dietary trial testing. This could potentially aid in discriminating between the dogs belonging to group 3, "questionable atopic".

The method of offending allergen identification was summarized for individuals from which this information was available. The software package Stata 8 was used for all analysis (Stata Corporation, College Station, Texas, USA).

## Results

### Study population

In the insurance database, a total of 373 individuals had at least one reimbursed claim for CAD during 2002. The claim file was missing in the insurance company's archive for 22 individuals, hence it was not possible to determine which veterinarian had treated these animals. Attempts were made to collect medical records from the attending veterinarian unless a full medical record was kept in the claims archive. In total, records were retrieved from 335 dogs (89.8%) and 63 practices submitted records to the study. The number of records obtained from each veterinary practice ranged from 1 to 40, with a median of 12.

### Descriptive statistics

**Figure 1:**
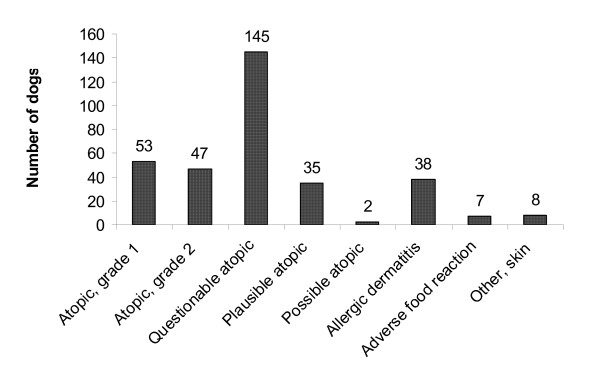
Re-classification of 335 Swedish dogs with a reimbursed insurance claim for CAD during 2002, based upon the criteria listed in table 1.

All 335 dogs included in the analysis were coded with CAD as the diagnosis in the insurance database. See figure 1 for a breakdown of the re-classified diagnoses based upon the available information from the medical records. If groups 1 to 5 were all considered CAD positive, a liberal 282 individuals (84.2%) would be re-classified as atopic. Alternatively, by assuming that all dogs in group 3 were CAD negative, the number re-classified as truly atopic would have been conservatively estimated to 137 individuals (40.9%).

The clinical signs documented in the medical records of the 109 dogs that had undergone both allergen testing and a diet trial are presented in table 2. Based upon the chi-squared test, no significant difference (p < 0.05) in the occurrence of recorded clinical signs existed between individuals with no response to the diet trial (groups 1 and 5) and those with partial (= reduction in clinical signs without complete remission) (group 2) or complete (group 7) response to such testing. The exception being that the presence of gastro-intestinal signs was more common in the latter group. Of the dogs with no response to diet testing (groups 1 and 5), 26 (47.3%) were male and 29 (52.7%) were female. Among the CAD and/or AFR dogs (groups 2 and 7), 30 (55.6%) were males and 24 (44.4%) females. A chi-square test revealed that these differences lacked statistical significance (p = 0.39). There were 42 different breeds represented in this subset. The most common breeds were German shepherd (n = 16), Labrador retriever (n = 10), West highland white terrier (n = 10), golden retriever (n = 9), mongrel (n = 5), dachshunds (n = 4) and English springer spaniel (n = 4). No association between breed and likelihood of responding to a diet trial was detected in this sample. The age at debut of clinical signs associated with CAD ranged from 3 to 48 months with a mean of 17 and median of 12 months, among the 52 dogs with no response to diet testing from which this information was available. Information on debut age in months was available for 52 dogs who responded to the diet trial, and it ranged from 2 to 53 with a mean of 16 and median of 12. Of the 109 animals in this subset, 64 (59.3%) were fed a commercial restricted protein diet, 33 (30.6%) a home-cooked diet, 8 (7.4%) a hydrolyzed protein diet and 4 (3.7%) an unknown test diet in order to rule out AFR. The chi-squared test revealed no effect of the type of diet on the outcome of the trial (p = 0.36).

**Table 2:** Clinical signs in 109 Swedish dogs with at least one reimbursed insurance claim for canine atopic dermatitis during 2002. The number and proportion of dogs displaying each clinical sign is summarized by response to dietary trials for elimination of adverse food reactions.

Clinical sign	No response to diet trial (n = 55)		Response to diet trial (n = 54)	
	Number	%	Number	%
Facial pruritus	18	32.7	14	26.0
Paw pruritus	30	54.6	34	63.0
Pruritic otitis	35	63.6	33	61.1
Pruritic ventral dermatitis	30	54.6	33	61.1
Pruritic generalized dermatitis	11	20.0	9	16.7
Interdigital erythema, bilateral	36	65.5	35	64.8
Debut before three years of age	47	85.5	50	92.6
Peri- oral, ophthalmic or otic erythema	28	50.9	34	63.0
Recurring dermatitis for more than two years	15	27.3	13	24.1
Family history of allergy	2	3.6	3	5.6
Relapse when humid	3	5.5	3	5.6
Rhinitis	4	7.3	3	5.6
Urticaria	0	0	2	3.7
Acral lick granuloma	4	7.3	2	3.7
Hyperhidrosis	1	1.8	0	0
Lichenification, flexor surfaces	2	3.6	2	3.7
Tonsilitis	7	12.7	2	3.7
Perineal pruritus	18	32.7	17	31.5
Inflammation/impaction of anal sack glands	5	9.1	3	5.6
Conjunctivitis	11	20.0	15	27.8
Gastrointestinal signs*	11	20.0	21	38.9

*chi-squared test significant at p < 0.05 level

The method of allergen identification was recorded for 250 individuals belonging to groups 1 to 5. Intradermal testing was the most commonly applied methodology and used in 227 (90.8%) of the cases, serology testing for specific IgE was used in 22 (8.8%) cases. In one single individual, both the tests were applied. The most commonly encountered positive test results were against house-dust mites; of the 282 individuals belonging to groups 1 to 5 192 (68.1%) had tested positive against *Dermatophagoides farinae*, 157 (55.7%) against *Tyrophagus putrescentiae*, 131 (46.5%) against *Acarus siro*, 64 (22.7%) against *Lepidoglyphus destructor *and 43 (15.3%) against *Dermatophagoides pteronyssinus*. Other, less commonly observed, reactions were against various pollens (26 dogs), down or feathers (20 dogs), sheep wool (9 dogs) and *Cladosporium herbarum *(9 dogs).

## Discussion

A previous study was performed utilizing a Swedish insurance database to estimate the incidence of and risk factors for canine atopic dermatitis [15]. Before any inferences can be drawn about the target population of insured Swedish dogs, or an even broader external population, one must assure that the internal validity of a study is acceptable [19]. The purpose of the current analysis was to validate the diagnosis of CAD in the insurance database against medical records. The next step in assuring the accuracy of the diagnosis in the insurance database would be to validate the claims records versus clinical observations performed by a diplomate in veterinary dermatology. However, this was not feasible due to the retrospective nature of the study.

The design of the study was cross-sectional, because all individuals were selected at the same time and subsequently re-classified by the principal investigator. All dogs in the study were originally coded with CAD in the insurance database. When comparing groups in this material it must therefore be kept in mind that the non-affected individuals do not represent a background population of healthy dogs but rather dogs with a very similar clinical presentation as the affected. The number of animals classified as having AFR only is low (n = 7), but does not represent the true proportion of food allergy versus atopy in the dog because the study sample only included individuals considered atopic by the treating veterinarian. Due to the nature of this study, the only available information about clinical presentation is what was recorded in the submitted medical records.

The results indicate that the accuracy of the diagnosis against clinical records was acceptable. None of the dogs included in the sample suffered from non-skin related disease, and the majority (97.6%) could be considered to have some allergic skin disease (groups 1 to 7). It should be noted that AFR had not been ruled out in the 145 dogs classified as "Questionable atopic" and that information about diet-testing was missing for the 35 dogs belonging to the group "Plausible atopic". Also, the 38 animals re-classified as having "allergic dermatitis" could potentially have been re-classified as CAD positive had they gone through a full work-up.

If all dogs classified as "Questionable atopic" in the current study were diet-tested without response, an optimistic estimate would be that 84.2% of the reimbursed cases could be re-classified as CAD positive. The most conservative estimate would be based on the assumption that these dogs all went into complete remission on the elimination diet and hence only 40.9% of the cases could be re-classified as CAD positive. Loeffler and others (2004) [20] showed that 20% of 46 pruritic dogs fed an elimination diet based on hydrolyzed protein responded completely and were judged as having AFR alone. If a similar result was to be obtained for the 145 dogs belonging to the group "questionable atopic" in the current sample, around 75% of the dogs in the study could be assumed to be atopic or have a dual diagnosis of atopy and AFR. Additionally, if the 38 dogs in the group "allergic dermatitis" would respond to diet trials to the same extent this estimate could be even higher.

Of a subset of the dogs in the current study that underwent a diet trial (n = 109), 55 (50.5%) showed no response ("Atopic grade 1" + "Possible atopic") while 54 (49.5%) responded partially ("Atopic grade 2") or fully ("Adverse food reaction"). The majority of these cases were fed a commercial test diet. However, the duration of diet trials was often unknown as well as any response to provocation. The attempt to use the clinical presentation in these somewhat better characterized patients to predict which animals in the group "questionable atopic" were likely to respond to a diet trial was unsuccessful. The only significant difference in recorded clinical presentation observed between individuals that responded to diet trials and those who did not was the presence of gastrointestinal signs, which was recorded for 21 (38.9%) and 11 (20.0%) of the dogs, respectively.

The method of allergen identification and most commonly encountered allergen reactions was presented for individuals from which such information was available. Most positive reactions against specific allergens were reported for different house-dust mites, while fewer dogs had positive reactions towards pollens. This observation is in accordance with a previous study performed in Sweden [21]. Low-cost treatments will often not be recorded in the insurance database because of the size of the deductible, and this will contribute to an under-estimation of CAD among patients that do not undergo expensive procedures such as intradermal testing for allergen identification or allergen specific immunotherapy. This may also be a contributing factor to why 90% of the recorded tests for identification of specific allergens in this material were performed using the intradermal methodology, as serology testing might cost less and hence not be recorded. The authors are under the impression that the incidence of CAD will be under-estimated in the insured population both because the cost of veterinary care for dermatology complaints may not exceed the deductible and because the ability of veterinarians to correctly diagnose the disease varies greatly. According to the definition by Jordan et al. (2004) the correctness of the diagnosis CAD was acceptable, but the completeness questionable in the investigated insurance database.

The response rate in the current study can be considered very good and information was available for 89.8% of the individuals that were diagnosed with CAD and reimbursed for the cost of this during 2002. Of the 373 dogs included in the original study sample, further information was not obtained from 22 because of missing claims records and 16 because of non-responders among veterinarians. Because the submitting veterinarians were aware that their records would be scrutinized by an external researcher, one could speculate that poorly documented cases would be with-held and only "high quality" records included. However, with very few records missing this potential source of bias should be of minor concern in the current study sample. Also, the veterinarians did not know what specific diagnosis was being investigated and hence should not be any less likely to submit "dubious" CAD cases. Another potential source of bias in the study is that only one investigator read and re-classified the submitted records. However, this was the most feasible strategy and the re-classification criteria were strictly adhered to in order to maintain objectivity.

## Conclusion

Overall, the agreement between the recorded diagnosis in the insurance database and the collected medical records was considered acceptable. However, it appears that many practitioners diagnose CAD without ruling out AFR hence for a large group of dogs it could not be determined whether or not they were truly atopic.

## Sammendrag

"Atopisk dermatitt hos hund: validering av registrert diagnose mot kliniske journaler fra 335 forsikrede hunder i Sverige"

Målet med studien var å undersøke hvorvidt hunder som er registrert med diagnosen atopisk dermatitt (AD) i en svensk forsikringsdatabase, faktisk har sykdommen. Fullstendige journaler fra alle hunder med et forsikringskrav for AD i 2002 ble forsøkt innhentet fra behandlende veterinær. Svarsprosenten var på 90%. Kliniske funn og diagnostiske tester som var notert i journalen ble sammenlignet med retningslinjene for å stille diagnosen AD. Overensstemmelsen var akseptablel, men mange hunder var ikke fullstendig utredet med hensyn på fôrintoleranse. Det var derfor ikke mulig å sikkert fastsette hvor stor andel av de akuelle hundene som faktisk var atopikere basert på det tilgjengelige materialet, men et estimat på rundt 75% blir foreslått.
